# Development and Validation of an ADA-Tolerant Assay for Quantification of an Exatecan-Based ADC in Monkey Plasma

**DOI:** 10.3390/molecules29030572

**Published:** 2024-01-24

**Authors:** Yimin Tao, Wei Lu, Jinli Gao, Shuangshuang Yang, Chaoyi Ruan, Yingying Hou, Jing Lu, Junjiu Xu, Jianjian Zhang, Stephanie Pasas-Farmer, Qiuping Qin, Likun Gong

**Affiliations:** 1 State Key Laboratory of Drug Research, Shanghai Institute of Materia Medica, Chinese Academy of Sciences, Shanghai 201203, China; ymtao@cdser.simm.ac.cn (Y.T.); jlgao@cdser.simm.ac.cn (J.G.); ssyang@cdser.simm.ac.cn (S.Y.); cyruan@cdser.simm.ac.cn (C.R.); yyhou@cdser.simm.ac.cn (Y.H.); jlu@cdser.simm.ac.cn (J.L.); jjxu@cdser.simm.ac.cn (J.X.); 2 Center for Drug Safety Evaluation and Research, Shanghai Institute of Materia Medica, Chinese Academy of Sciences, Shanghai 201203, China; 3 OnCusp Therapeutics, New York, NY 10013, USA; wei.lu@oncusptx.com; 4 Multitude Therapeutics Inc., Shanghai 200233, China; jianjian.zhang@multitudetherapeutics.com; 5 BioData Solutions LLC, Lawrence, KS 66044, USA; spfarmer@bdatasolutions.com; 6 University of Chinese Academy of Sciences, Beijing 101408, China; 7 Zhongshan Institute for Drug Discovery, Shanghai Institute of Materia Medica, Zhongshan 528400, China

**Keywords:** antibody–drug conjugate (ADC), bioanalysis, anti-drug antibody (ADA), ligand binding assay (LBA), ADA-tolerant assay

## Abstract

Background: The development of an anti-drug antibody (ADA)-tolerant pharmacokinetic (PK) assay is important when the drug exposure is irrelevant to toxicity in the presence of ADA. We aimed to develop and validate an ADA-tolerant assay for an exatecan-based antibody–drug conjugate (ADC) in monkey plasma. Results: The assay tolerated 5.00 µg/mL of ADA at 12 µg/mL of ADC. Its accuracy and precision results satisfied the acceptance criteria. Furthermore, the assay was free from hook and matrix effects and exhibited good dilutional linearity. Additionally, the ADC in plasma samples was stable under different storage conditions. Method: An ADA-tolerant ADC assay was configured with an anti-payload antibody for capture, and a drug-target protein combined with a horseradish peroxidase (HRP)-labeled antibody against a drug-target-protein tag for detection. Samples were firstly acidified to dissociate drug and ADA complexes, and to convert the carboxylate form to the lactone form of exatecan molecules; then, the ADAs in the samples were removed with a naked antibody-coated microplate. The treated samples were further incubated with coated anti-payload antibody and captured ADC molecules were quantified by the detection reagent. The developed assay was optimized and validated against regulatory guidelines. Conclusions: The assay met both methodological and sample-related ADA tolerance requirements, and was applicable to a nonclinical study in cynomolgus monkeys.

## 1. Introduction

Antibody–drug conjugates (ADCs) are a class of novel therapeutics for cancer, which consist of a tumor-targeting monoclonal antibody (mAbs) conjugated to a cytotoxic payload using a chemical linker. Since the first ADC was approved in 2000 by the US Food and Drug Administration, 14 ADCs have been approved worldwide and more than 100 candidates are being investigated in clinical stages at present [1]. Meanwhile, the continued advances in ADC components, including antibody engineering, site-specific conjugation, and novel cytotoxic drugs, have promoted the development of various ADC platforms. However, the bioanalysis of ADCs has been faced with challenges due to their complexity derived from the transformation and catabolism of ADCs in vivo. The major analytes for ADCs in pre-clinical and clinical studies include total antibody, conjugated antibody (total ADC), and free payload. There are a number of factors that should be taken into consideration during the development of bioanalytical methods for ADCs. First, an appropriate assay format should be selected for the analyte of interest. Second, the assays for total antibody and conjugated antibody should ideally be unaffected by the drug-to-antibody ratio (DAR) [2,3,4,5,6]. DAR is an important parameter reflecting the number of payloads conjugated to the antibody, and its change over time indicates ADC deconjugation and other biotransformation processes [6]. Third, whether the structure of the payload linked to the antibody undergoes some changes in vivo [7]. For example, camptothecin-based ADCs can undergo a conversion from the lactone ring structure of camptothecins to the carboxylate structure of camptothecins or vice versa, depending upon their pH condition [8]. Such changes, in turn, also cause heterogeneity in the relevant ADC. Last, the extent to which the assay is affected by anti-drug antibodies (ADAs) in the sample. ADAs can interfere with the pharmacokinetic (PK) assay used to measure the drug concentration in circulation, thereby impacting PK or toxicokinetic (TK) calculations and drug exposure profiles.

ADAs can impact the quantification of ADCs by interfering with either capture or detection reagents in ligand binding assays as well as the capture and digestion reagents in hybrid LC-MS/MS methods [9,10]. Consequently, high-level ADAs may lead to altered PK profiles due to ADA-mediated assay interference [11]. In other words, a compromised PK assessment will likely generate an underestimation of bioactive drugs that may cause an increased risk to patients. In addition, PK profiles unmatched to efficacy responses also pose difficulties in data interpretation. Therefore, the development of an ADA-tolerant PK method is warranted if the drug levels do not correlate with pharmacodynamic (PD), safety, and/or efficacy data [11].

Here, we report the development and validation of an ADA-tolerant ligand binding assay for an exatecan-based ADC named *SM22-64-02* with a DAR value of 8. Along with an acid pretreatment step performed for the samples, controls, and standards to dissociate drug and anti-drug antibody complexes, and simultaneously convert the carboxylate form to the lactone form of exatecan molecules, ADAs in the samples were removed by using a naked antibody-coated microplate, and consequently, the potential effect caused by ADAs on the assay was mitigated. The samples treated as described above were further incubated with an anti-payload antibody-coated microplate and captured ADC molecules were quantified by the detection reagent. The assay was thereafter validated against regulatory guidelines for its performance in a GLP-compliant environment, thereby establishing its suitability for application to a nonclinical toxicology study in cynomolgus monkeys. Furthermore, it should be noted that the strategy of using a naked antibody to remove ADAs worked well for this ADC and could also be used in other cases where the ADC itself cannot be used as an immune-affinity capture substance due to high background signals obtained.

## 2. Results

### 2.1. Method Development

#### 2.1.1. DAR-Insensitive Assay Development

**Figure 1:**
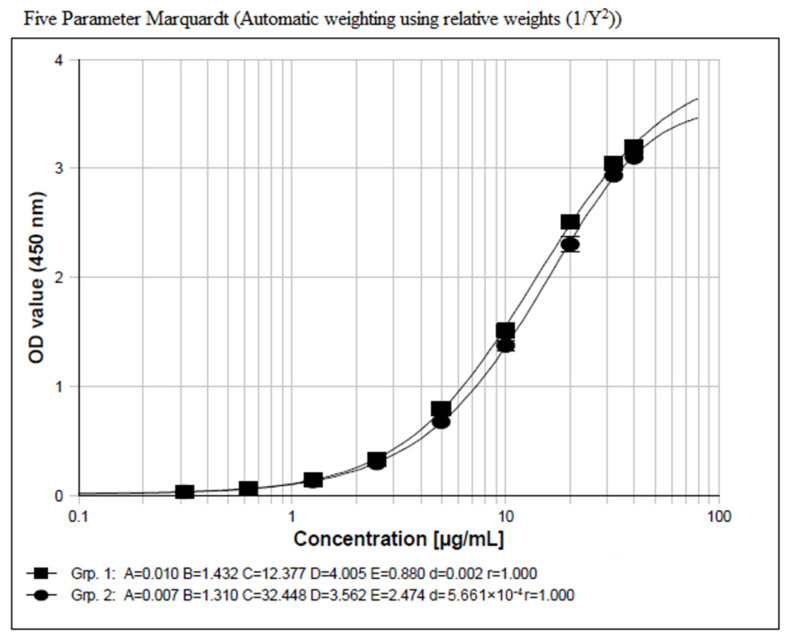
Concentration–signal response relationship of an ADC with a DAR value of 8 (solid rectangle line) versus that of an ADC with a DAR value of 4 (solid oval line). The x-axis represents nominal concentrations of calibration standards and the y-axis represents optical density (OD) values at 450 nm after subtraction of a matrix blank signal. A logistic model (5-parameter weight 1/Y^2^) is used for curve fitting with the following equation: Resp. = (A − D)/((1 + (Conc./C) ^B^) ^E^) + D. Among the parameters, A, B, C, D, and E are values for different coefficients, d stands for average square deviation of coefficient values, and r stands for the absolute value of the correlation coefficient.

Due to in vivo catabolism, a study sample may contain a complex ADC mixture with changing DARs. Thus, the calibration standard curve comprised of a reference standard with the starting DAR would not represent the ADC with changing DARs in different samples [3]. To solve this problem, capture and detection reagents should be carefully selected to overcome or reduce the DAR-induced bias in ADC bioanalysis. Several detection reagents were selected during the method development stage. Finally, a DAR-insensitive method was developed with an assay configured with an anti-payload antibody for capture and a mixture of His-tagged antigen and HRP-labeled anti-His-antibody for detection. As shown in Figure 1, the calibration curve of the ADC with a DAR value of 8 was very close to that of the ADC with a DAR value of 4, with OD ratios between the two ADCs ranging from 1.00 to 1.17 at tested concentration points. The above results indicated that both the capture reagent and the detection reagent used in the comparison experiment were basically not affected by the different DARs. Thus, the reagents were picked for subsequent use in the assay construction.

#### 2.1.2. Lactone-Insensitive Assay Development

*SM22-64-02* is an exatecan containing ADC. Exatecan is one of the antineoplastic camptothecin derivatives which exists in a pH-dependent equilibrium between the open carboxylate and the closed lactone forms. This kind of transformation may lead to differences in terms of the ratio of the two molecular forms in different samples as well as in standards, leading to inaccurate ADC results. Apart from that, the anti-payload antibody used may react differently with the two forms, which will also generate inaccurate ADC results. To resolve this issue, we incorporated an acid pretreatment step by using 0.3 M acetic acid in the assay because the carboxylate form of camptothecin can turn into a closed lactone form in an acid environment [12]. As a result, all the carboxylate form exatecan molecules in both matrix and standard samples were converted to the lactone form of exatecan molecules before proceeding to the capture and detection steps of the assay. Meanwhile, the pretreatment step also prevented the anti-payload antibody used in the assay from reacting with the two forms of exatecan molecules, thereby ensuring that accurate results were obtained with the assay.

#### 2.1.3. ADA-Tolerant Assay Development

**Figure 2:**
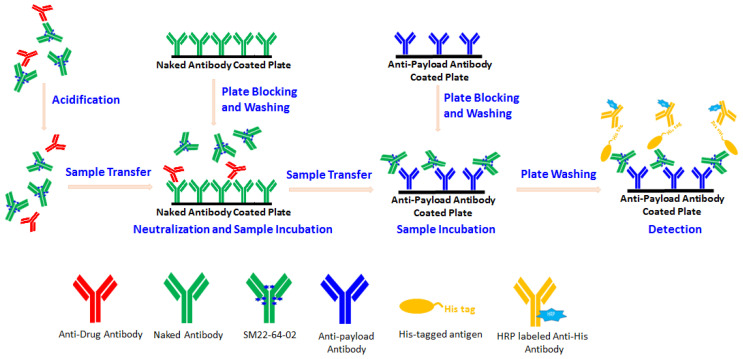
Schematic diagram of the present ADA-tolerant ADC assay. For assay configuration, the test sample containing the exatecan-based ADC and ADAs is first acidified. The ADAs are then removed by immune-affinity binding to the naked antibody coated on a microplate after the neutralization of acid-treated samples. Thereafter, the exatecan-based ADC molecules are captured by an anti-payload antibody coated on another microplate and measured with the detection reagent composed of His-tagged antigen and HRP-labeled anti-His antibody.

In addition, the ADC assay to be developed for *SM22-64-02* must be ADA-tolerant to avoid underestimation of exposure during preclinical sample analysis. The aforementioned acidification treatment of the samples can convert the carboxylate form of the exatecan molecules to the lactone form of the exatecan molecules, as well as dissociate ADA–ADC complexes and liberate the drug. To make the assay tolerate the ADAs in the samples, we added a solid phase adsorption step to the assay via the use of a naked antibody-coated microplate. The acidified samples were first transferred to the naked antibody-coated microplate and immediately neutralized there; free ADAs were then able to bind to the solid-phased naked antibody, and the supernatant was transferred to another microplate where the anti-payload antibody was coated on each well. After incubation, the detection reagent was added and reacted with the ADC bound to the well surface of the microplate. The microplate was thereafter washed and TMB was added to each well for color development, which was terminated with 2 M H_2_SO_4_. The OD value at 450 nm was measured with a microplate reader and the conversion of OD values for test samples into concentrations of ADC was performed using a software-mediated comparison to a concurrently analyzed standard curve regressed according to a five-parameter logistic model with the weight 1/Y^2^ (see Figure 2).

To understand the ADA impact on the ADC assay, we prepared the following test samples during the assay development: monkey plasma samples containing *SM22-64-02* at the concentrations of LLOQ (1.25 µg/mL), LQC (3.00 µg/mL), MQC (12.0 µg/mL), HQC (22.5 µg/mL), and ULOQ (30.0 µg/mL) were spiked with ADA to reach concentrations of 0.250 µg/mL, 1.00 µg/mL, and 5.00 µg/mL, respectively. These samples were treated with or without the ADA removal procedure, which means that the test samples after acidification were neutralized in the naked antibody-coated microplate with the ADA removal procedure or neutralized in the anti-payload antibody-coated microplate directly without the ADA-removal procedure.

The results showed that the measurement of *SM22-64-02* without the ADA removal procedure would be impacted at the LLOQ level when the concentration of ADA in the sample was from 0.250 μg/mL to 1.00 μg/mL. Moreover, it was fully impacted when the concentration of ADA reached 5.00 μg/mL (Table 1). However, when neutralized in a naked antibody-coated plate, the anti-drug antibodies were removed and the assay of *SM22-64-02* was fully tolerant to ADA with a concentration of up to 1.00 µg/mL. And the quantification of *SM22-64-02* was accurate at the MQC level when the concentration of ADA reached 5.00 μg/mL (Table 1). These results indicated that the removal of ADA by the naked antibody-coated microplate could markedly enhance ADA tolerance in the ADC assay.

### 2.2. Method Validation

After the assay concept of the ADA-tolerant ADC assay was approved, the conditions of the prototype assay were optimized. Thereafter, the optimized assay was validated against the relevant regulatory guidelines [13,14,15].

#### 2.2.1. Calibration Curve

**Figure 3:**
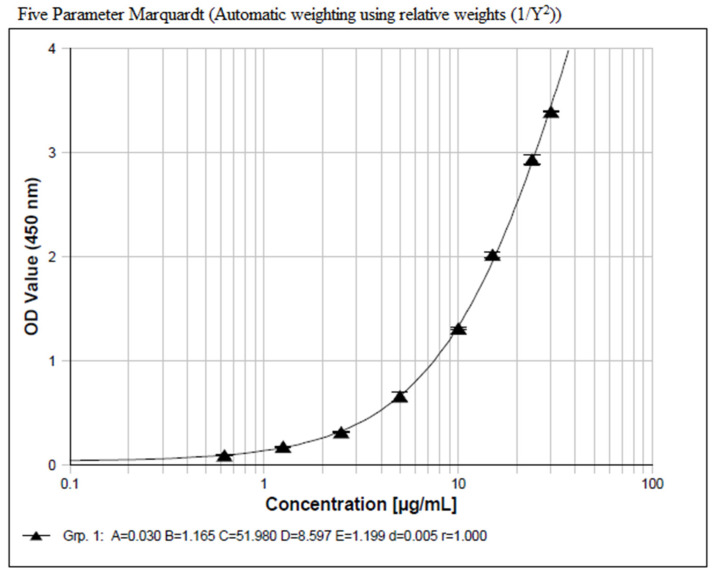
Typical calibration curve of the ADC assay. The x-axis represents nominal concentrations of calibration standards and the y-axis represents instrument responses (OD value at 450 nm) after subtraction of a matrix blank signal.

Seven different non-zero standards with an analytical range from 1.25 µg/mL to 30.0 µg/mL and a lower anchor point of 0.625 µg/mL prepared in a pooled monkey K_2_EDTA plasma were used to construct the calibration curve. The concentration–response relationship was fitted to a five-parameter logistic model with the weight of 1/Y^2^. All calibration curves obtained during the validation met the acceptance criteria shown in Supplementary Table S1. The typical calibration curve is given in Figure 3.

#### 2.2.2. Accuracy and Precision

**Figure 4:**
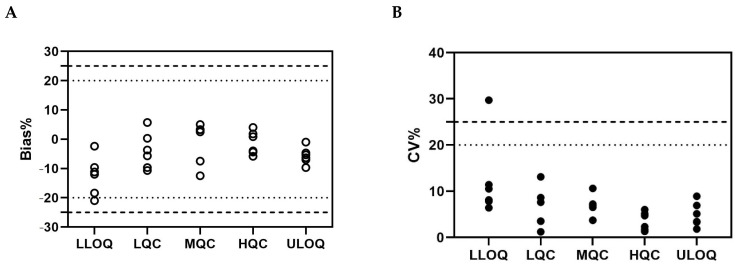
Intra-assay accuracy and precision of the ADC assay. The % bias and % CV of different QC samples from six independent intra-assay accuracy and precision runs are presented, respectively. The dotted lines and dashed lines shown in (**A**) represent a ±20% range for the LQC, MQC, and HQC, and a ±25% range for the LLOQ and ULOQ, respectively. The dotted line and dashed lines shown in (**B**) represent the 20% CV limit for the LQC, MQC, and HQC, and the 25% CV limit for the LLOQ and ULOQ, respectively. Hollow and filled circles represent Bias % and CV %, respectively.

Six runs to determine accuracy and precision were performed using five levels of QC samples (ULOQ, HQC, MQC, LQC, and LLOQ) by four analysts over 5 days. The % bias and % CV of six independent intra-assay accuracy and precision runs are given in Figure 4, among which five out of six intra-assay accuracy and precision runs met the acceptance criteria shown in Supplementary Table S1, and the % CV of LLOQ from one batch was beyond 25.0%. The results of inter-assay accuracy and precision given in Table 2 also met the acceptance criteria shown in Supplementary Table S1.

#### 2.2.3. Assay Selectivity

**Figure 5:**
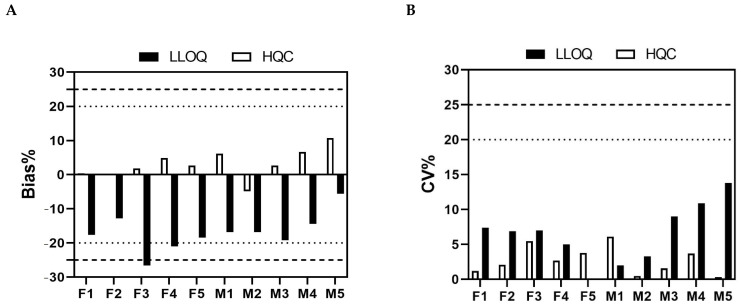
Selectivity evaluation. Ten lots of naive plasma samples (five females and five males) were used for selectivity evaluation at HQC and LLOQ levels. F1 to F5 stand for female 1 to female 5 plasma samples, and M1 to M5 stand for male 1 to male 5 plasma samples. The results indicated that 9/10 of individual test samples met the acceptance criteria, except that the bias % of female 3 (F3) at the LLOQ level was slightly beyond −25.0%. The dotted lines and dashed lines shown in (**A**) represent ±20% and ±25% ranges for the HQC and LLOQ, respectively. The dotted line and dashed lines shown in (**B**) represent 20% and 25% CV limits for the HQC and LLOQ, respectively.

Selectivity is the ability of a method to differentiate and quantify the analyte of interest in the presence of non-specific matrix components. Selectivity testing results indicated that 90% (9/10) of individual test samples met the acceptance criteria shown in Supplementary Table S1 (Figure 5). In addition, all unspiked test samples generated lower-than-related LLOQ results.

#### 2.2.4. Hook Effect and Dilution Linearity

The hook effect testing results shown in Table 3 were all above the qualification limit (AQL), indicating that no hook effect was observed when the concentration of *SM22-64-02* reached 1000 µg/mL. The results of the dilution linearity test given in Table 3 also showed that *SM22-64-02* could be accurately measured after 10- to 200-fold dilutions in cynomolgus monkey K_2_EDTA plasma with the bias % ranging from −20.0% to −6.0% and CV % from 2.8% to 8.9%.

#### 2.2.5. Stability

**Figure 6:**
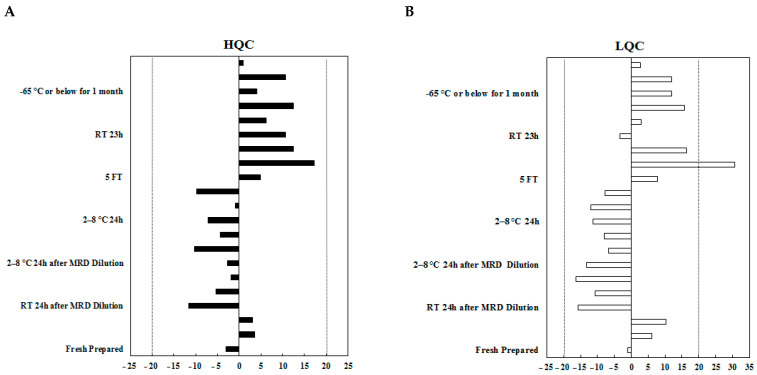
Evaluation of different stability conditions. LQC- and HQC-level samples stored at different conditions were used for stability evaluation. Each set of stability test samples included an LQC- and an HQC-level sample. For each storage condition, three sets of stability test samples were analyzed, respectively. The results of stability testing under different conditions all met the acceptance criteria shown in Supplementary Table S1. The dotted lines represent ±20% ranges for the LQC-level samples in (**A**) and for the HQC-level samples in (**B**).

Stability evaluation was carried out to ensure that every step taken during sample preparation, processing, and analysis as well as storage conditions would not impact the concentration of the analyte. The results of stability testing are shown in Figure 6. The stability results met the acceptance criteria shown in Supplementary Table S1, indicating that *SM22-64-02* was stable at the tested conditions.

## 3. Materials and Methods

### 3.1. Reagents and Disposables

The ADC (*SM22-64-02*, DAR = 8), conjugated antibody with a DAR value of 4 (DAR4 ADC), and related naked antibody (mAb) were provided by Wuxi Biologics (Wuxi, China). Mouse anti-payload monoclonal antibody was provided by Abmart Pharmaceutical Technology (Shanghai) Co. Ltd. (Shanghai, China). His-tagged antigen was purchased from Acro Biosystem (Beijing, China). HRP-tagged anti-His antibody (cat. no. A00612) was purchased from GenScript (Piscataway, NJ, USA). The positive anti-drug antibody, provided by OnCusp Therapeutics (New York, NY, USA), was a purified polyclonal antibody against *SM22-64-02* raised from a rabbit. Bovine serum albumin (BSA) (cat. no. A500023) was purchased from Sangon Biotech (Shanghai, China). TMB was purchased from KPL (cat. no. 5120-0077). The 1 × PBS, pH7.2 was purchased from Thermo Fisher Scientific (Waltham, MA, USA) (cat. no. 20012). Acetic acid (cat. no: 10000218), Tris (hydroxymethyl) aminomethane (cat. no. 30188360), concentrated hydrochloric acid (cat. no. 10011018), and concentrated sulfuric acid (cat. no. 10021618) were purchased from Sinopharm Co Ltd. (Beijing, China) Nunc F96 microplates (cat. no. 439454) were purchased from ThermoFisher Scientific. Naȉve cynomolgus monkey K_2_EDTA plasmas (NPP) and other in-house-made solutions or buffers were supplied by the Centre for Drug Safety Evaluation and Research (CDSER), Shanghai Institute of Materia Medica (SIMM), (Shanghai, China). The pooled naïve cynomolgus monkey plasma (PNPP) was made by mixing plasma samples from at least 10 different animals.

### 3.2. Assay Procedure

For the assay with the ADA-removal procedure, two Nunc F96 microplates were pre-coated with the naked antibody (10 µg/mL, 200 μL/well) and anti-payload antibody (2 µg/mL, 100 μL/well), respectively, and the plates were incubated at 2–8 °C, overnight. After washing with PBST (0.01 M PBS with 0.05% Tween 20), the microplates were blocked with 3% BSA-PBST. Meanwhile, 25 μL of 20-fold (MRD) diluted samples were acidified with 225 μL of 0.3 M acetic acid in a new NUNC dilution plate and incubated at room temperature for 1 h on a plate shaker. Subsequently, 60 μL of 1 M Tris-HCl (pH9.0) and 140 μL of samples after acidification were transferred to the naked antibody-coated microplate and incubated at 37 °C for 1 h. After neutralization, 70 μL of samples and 30 μL of 1% BSA-PBST were added to the anti-payload antibody-coated microplate and incubated at 37 °C for 2 h. After washing, 100 μL of the detection solution containing His-tagged antigen and anti-His antibody in 1% BSA-PBST was added and incubated at 37 °C for 2 h. After further washing, 100 µL of TMB was added to the microplate, followed by adding 50 µL of stop solution. The plate was read using a Tecan Sunrise plate reader at 450 nm.

For the assay without the ADA-removal procedure, the acidified samples were neutralized directly in an anti-payload antibody-coated plate. Other steps were the same as for the assay with the ADA-removal procedure.

### 3.3. ADA Interference

The monkey plasma samples containing *SM22-64-02* at the concentrations of LLOQ (1.25 µg/mL), LQC (3.00 µg/mL), MQC (12.0 µg/mL), HQC (22.5 µg/mL), and ULOQ (30.0 µg/mL) were spiked with the polyclonal ADA to reach concentrations of 0.250 µg/mL, 1.00 µg/mL, and 5.00 µg/mL, respectively. These mock samples were used for the evaluation of ADA impact on the PK assay with or without the ADA-removal procedure.

### 3.4. Method Validation

The method validation was performed according to the US FDA guidance, European Medicines Agency (EMA) guidelines, and ICH M10 for bioanalytical methods validation [13,14,15]. The method validation experiments included calibration curve, accuracy and precision, selectivity, hook effect, dilution linearity, and stability. The acceptance criteria of each test item are presented in Supplementary Table S1.

#### 3.4.1. Calibration Curve

The calibration curve contained the following nominal concentration points: 30.0 μg/mL, 24.0 μg/mL, 15.0 μg/mL, 10.0 μg/mL, 5.00 μg/mL, 2.50 μg/mL, 1.25 μg/mL, and 0.625 μg/mL, among which 30.0 μg/mL and 1.25 μg/mL were ULOQ and LLOQ, respectively, and 0.625 μg/mL was the lower anchor point. The PNPP served as a matrix blank.

#### 3.4.2. Accuracy and Precision

For the intra-assay accuracy and precision experiments, ULOQ, HQC, MQC, LQC, and LLOQ samples were tested in three sets on the same plate. For the inter-assay accuracy and precision experiment, six independent runs were performed by four analysts during five days. Every sample within each set was analyzed in duplicate.

#### 3.4.3. Selectivity

Plasma samples from 10 different individuals (the number of males being the same as that of females) were used to prepare selectivity test samples. Each plasma sample was divided into three sets and spiked with *SM22-64-02* at the concentrations of HQC and LLOQ. In addition, the individual sample not containing *SM22-64-02* (0 μg/mL) was also tested.

#### 3.4.4. Hook Effect and Dilution Linearity

*SM22-64-02* was spiked into a neat matrix to prepare hook effect test samples at the concentrations of 125 µg/mL, 250 µg/mL, 500 µg/mL, 800 µg/mL, and 1000 µg/mL. The hook effect test samples were analyzed after MRD with 1% BSA-PBST solution. The dilution linearity samples were the same samples but evaluated in a different way, as follows: prior to analysis, the samples were first treated for MRD with 1% BSA-PBST solution and then 10-, 20-, 50-, 100-, and 200-fold diluted with 1% BSA-PBST solution containing 5% PNPP, respectively.

#### 3.4.5. Stability

Stability samples of HQC and LQC concentrations were prepared from *SM22-64-02* with PNPP. The freshly prepared samples, and aliquot samples stored at room temperature, 2–8 °C for 24 h, and −65 °C or lower for approximately 1 month were analyzed. Sample stability at room temperature and at 2–8 °C for 24 h after MRD dilution was also analyzed. For testing the stability of freeze/thaw treatment, high- and low-stability testing samples were stored at −65 °C or lower, and samples undergoing 5 freeze/thaw cycles were analyzed.

### 3.5. Software for Data Acquisition and Processing

The data were collected using a Magellan Tracker V7.2 (Tecan Trading AG, Männedorf, Switzerland), and a Watson LIMS 7.5 SP1 (Thermo Fisher Scientific, Waltham, MA, USA) was used for the calibration curve fitting (5-parameter logistic (Marquardt) model (weight 1/Y^2^)). Microsoft Excel 2007 (Microsoft, Redmond, WA, USA) was used for the calculation of bias %, CV %, and total error for accuracy and precision. All the graphs were generated using GraphPad Prism version 9.5.0 (GraphPad Software Inc., San Diego, CA, USA).

## 4. Discussion

The quantification of an ADC has been challenging because of its heterogeneous nature after drug administration. Generally, the reference analytical standard may not represent the ADC in vivo, especially in the elimination phase. There have been discussions aimed at resolving the issue, covering topics such as assay format, crucial reagents, and so on [2,3,4,5,6]. An ideal ADC quantification method should be DAR-insensitive, and thus the reference standard can be representative of the ADC forms with different DARs, and the measured concentrations reflect the actual ones, thereby yielding correct drug exposure data in vivo. In the present study, a conjugated antibody with a DAR value of 4 was used to evaluate the DAR impact on bioanalysis during the critical reagent selection phase of the method development. We found that the assay configured with the anti-payload antibody as the capture reagent and the mixture of His-tagged antigen and HRP-labeled anti-His-antibody as the detection reagent generated comparable binding signals over the concentration range from 1.25 μg/mL to 30.0 μg/mL, indicating that the assay is insensitive to DAR values from 4 to 8. As a conjugated antibody with DAR 2 was not available throughout the study, it was regretful that we could not evaluate the performance of the assay for the conjugated antibody with lower DAR values. However, our previous study indicated that *SM22-64-02* was quite stable in cynomolgus monkey K_2_EDTA plasma with scant release of exatecan molecules in vitro after 21 days of incubation at 37 °C. Furthermore, since DAR 8 and DAR 4 generated comparable binding signals with the assay, this fact indicates that both the anti-payload antibody and the detection reagent do not bind to the conjugation sites as well as their vicinities, and, therefore, also suggests that the assay would be most likely unaffected by the conjugated antibody with lower DAR values.

As a classic anti-tumor drug, camptothecins exist in a pH-dependent reversible equilibrium between the closed lactone and the open carboxylate forms. At lower pH, the equilibrium shifts toward the active lactone form [8,16,17,18,19]. This transformation may lead to differences in the ratio of the two molecular forms not only in different samples collected from the same subject at different time points but also in different samples collected from different subjects. Furthermore, this transformation may also lead to differences in the ratio of the two molecular forms between the blood samples and standard samples. Finally, the anti-payload antibodies used may react differently with the two forms of molecules, producing different binding signals. Therefore, all these factors contribute to the inaccurate concentration results of the ADC. However, there are reports on using two strategies to eliminate the lactone influence on the measurement of ADC concentrations. First, a covalent linkage occurring through the α-hydroxylactone position can stabilize the lactone ring [20,21], thus leaving only one molecular form, namely the lactone ring, present in the circulation, e.g., IMMU-132. Second, instead of stabilizing the lactone ring, an anti-camptothecin derivative antibody can be generated, e.g., T-Dxd, with the same performance on the lactone and carboxylate subtypes [22,23]. Unfortunately, the two mentioned strategies were unsuitable for the current ADC as it not only lacks a covalent linkage through the α-hydroxylactone position but also lacks an antibody with the same performance on the lactone and carboxylate subtypes.

The acidification treatment used for the samples, including standards and controls, is a necessary step for the assay [13]. It serves two purposes: First, it allows dissociation of drug molecules from anti-drug antibodies, and the liberated anti-drug antibodies can then be removed by immune-affinity binding with the naked antibody immobilized on the surface of a microplate. Second, it allows the conversion of the carboxylate form to the lactone form of exatecan molecules, and the influence stemming from different forms of exatecan molecules in the samples on the measurement of ADC concentrations can be eliminated. In this way, an ADA-tolerant and lactone-insensitive assay can be made possible.

It should be pointed out that quantification of ADC molecules regardless of their lactone or carboxylate form in individual samples is necessary. This is related to the mechanism of action of the ADC molecules in vivo. These ADC molecules are firstly internalized to tumor cells and then further brought to the lysosome where the acid environment will turn the open carboxylate form into the closed lactone form of exatecan molecules [8], thereby exerting the anti-tumor effect of the ADC. As such, there is no need to separately quantify the lactone form and the carboxylate form of exatecan molecules as the two forms are both biologically relevant species.

The development of an ADA-tolerant PK assay is useful when drug exposure in the samples with ADAs is not correlated with toxicity or efficacy data. ADAs can alter the clearance of the drug and consequently impact circulating drug levels, affecting the PK profile. Apart from that, ADAs may affect the analysis of the drug by inhibiting the binding of capture and detection reagents of the analytical assay to their specific analyte epitopes, usually generating underestimated drug levels [10,22]. Among ADAs, non-neutralizing antibodies might increase the risks in patients as the underestimated drug exposure is not consistent with toxicity response or PD biomarker data [11]. By using matrix samples spiked with both drug and positive anti-drug antibodies, we found the quantification of *SM22-64-02* with the current ADC assay to be accurate at the LLOQ level (1.25 µg/mL) when the concentration of ADA reached 1.00 μg/mL, and nearly unaffected by 5.00 μg/mL of ADA at the LQC level (3.00 µg/mL). Compared with the assay without the ADA removal procedure, the present assay provides an improved tolerance level for ADA in the study samples. Notably, considering the fact that the positive anti-drug antibody was generated from a hyperimmune rabbit, the tolerance levels obtained with such a positive antibody likely do not reflect the tolerance levels with ADAs from the real study samples. This means that the tolerance levels given in this paper are likely underestimated.

Although the current assay performance has met the acceptance criteria of the method validation, it is not without its limitations. Logically, the ADC rather than the naked antibody should have been used to coat the microplate to remove the ADAs from each sample during the neutralization phase. But we could not do it this way as the associated nonspecific binding signal was rather high. Consequently, those ADAs reacting with the non-antibody portion of the ADC could not be removed from the sample by the ADA-removal procedure mediated by the naked antibody. However, it was found that the ADAs generated in a preliminary nonclinical study were all bound to the antibody portion of the ADC. Therefore, such a limitation should have little impact on the results obtained with the current assay. Apart from that, the strategy of using a naked antibody to remove ADAs could also be used in other cases where the ADC itself cannot be used as an adsorbing substance.

## 5. Conclusions

Based on the acid dissociation and solid-phase ADA removal procedure, an ADA-tolerant ELISA was developed and validated for the first time for the quantification of an exatecan-based ADC in monkey K_2_EDTA plasma. The assay was free from lactone influence and its ADA tolerance level was markedly improved for the study samples. Furthermore, all the validated parameters satisfied the acceptance criteria, which are in line with the guidance requirements of the regulatory agencies such as the FDA, EMA, and ICH M10 for bioanalytical method validation [13,14,15]. Taken together, these features indicate that the present assay is fit for related study purposes. Moreover, the assay is particularly useful when drug exposure in the samples with ADAs is not correlated with toxicity or efficacy data.

## Figures and Tables

**Table 1:** Assay tolerance to anti-drug antibodies.

ADA Conc. (µg/mL)	*SM22-64-02* Nominal Conc. (µg/mL)	ADA Interference			After ADA Removal		
		Measured Conc. (µg/mL)	CV %	Bias %	Measured Conc. (µg/mL)	CV %	Bias %
5.00	30.0	*21.6*	*6.1*	*−28.0*	24.7	1.8	−17.7
	22.5	*17.0*	*4.6*	*−24.4*	18.8	0.0	−16.4
	12.0	*8.66*	*9.7*	*−27.8*	9.91	1.3	−17.4
	3.00	*1.28*	*35.8*	*−57.3*	*2.08*	*3.2*	*−30.2*
	1.25	*BQL*	*NA*	*NA*	*BQL*	*NA*	*NA*
1.00	30.0	29.5	6.9	−1.7	27.6	2.5	−8.0
	22.5	19.9	2.5	−11.6	20.8	0.8	−7.6
	12.0	10.7	0.2	−10.8	11.1	3.4	−7.5
	3.00	2.56	14.2	−14.7	2.86	2.3	−4.6
	1.25	*BQL*	*NA*	*NA*	1.05	5.7	−15.8
0.250	30.0	26.4	0.8	−12.0	28.3	0.0	−5.7
	22.5	19.6	6.4	−12.9	24.2	0.9	7.6
	12.0	10.4	1.3	−13.3	12.3	1.9	2.5
	3.00	2.58	5.4	−14.0	3.00	11.2	−0.1
	1.25	*BQL*	*NA*	*NA*	1.09	13.0	−12.9
0.00	30.0	28.1	7.2	−6.3	27.8	0.5	−7.3
	22.5	20.8	9.9	−7.6	22.1	2.6	−1.8
	12.0	11.5	2.5	−4.2	12.8	7.7	6.7
	3.00	2.76	8.5	−8.0	3.39	3.9	13.1
	1.25	1.16	17.8	−7.2	1.16	5.0	−7.4

NA stands for not available. Numbers or text in italics indicate the presence of ADA interference.

**Table 2:** Inter-assay accuracy and precision.

Item	LLOQ (1.25 µg/mL)	LQC (3.00 µg/mL)	MQC (12.0 µg/mL)	HQC (22.5 µg/mL)	ULOQ (30.0 µg/mL)
Inter-run Mean	1.09	2.88	11.9	22.2	28.3
Inter-run % CV	7.6	6.5	7.3	4.0	3.0
Inter-run % Bias	−12.8	−4.0	−0.8	−1.3	−5.7
Inter-run % Total Error	20.4	10.5	8.1	5.3	8.7
*n*	6	6	6	6	6

**Table 3:** Hook effect and dilution linearity.

Nominal Conc. (μg/mL)	Dilution Factor	Measured Conc. (μg/mL)	Dilution Factor	Measured Conc. (μg/mL)	Mean Conc. (μg/mL)	CV %	Bias %
1000	1	AQL	200	924	914	4.7	−8.6
		AQL		888			
		AQL		936			
		AQL		965			
		AQL		855			
800	1	AQL	100	656	658	5.8	−17.8
		AQL		657			
		AQL		681			
		AQL		598			
		AQL		698			
500	1	AQL	50	503	470	7.1	−6.0
		AQL		510			
		AQL		442			
		AQL		442			
		AQL		455			
250	1	AQL	20	214	222	2.8	−11.2
		AQL		218			
		AQL		223			
		AQL		225			
		AQL		230			
125	1	AQL	10	92.3	100	8.9	−20.0
		AQL		91.2			
		AQL		99.9			
		AQL		106			
		AQL		112			

## Data Availability

The original contributions presented in the study are included in the article/Supplementary Material, further in-quiries can be directed to the corresponding authors.

## Supplementary Materials

The following supporting information can be downloaded at: https://www.mdpi.com/article/10.3390/molecules29030572/s1, Table S1: Acceptance criteria of method validation.
