# Supplementary material for: Development and Validation of an ADA-Tolerant Assay for Quantification of an Exatecan-Based ADC in Monkey Plasma
Source: Molecules. 2024 Jan 24;29(3):572. doi: 10.3390/molecules29030572 (PMC10856772; doi:10.3390/molecules29030572)
Supplement: Supplementary file 1 [file molecules-29-00572-s001.zip › molecules-2719941-supplementary.pdf]

Supplementary Table S1. Acceptance Criteria of Method Validation

| Item | Acceptance Criteria |
| --- | --- |
| Calibration Curve | For each assay batch, the target back-calculated concentrations of the calibration standards (exclude anchor point) should be within ±20.0% of the nominal value (±25.0% at ULOQ, LLOQ) with the concentration CV% not exceeding 20.0% (25.0% at ULOQ, LLOQ). ≥75% and at least 6 non-zero calibration standard concentration points meet above criteria. The anchor point has no acceptance criteria. |
| Intra-assay accuracy and precision | The concentration Bias% based on the mean concentration of 3 sets of samples should be within ±20.0% of the nominal value at each concentration level (±25.0% at the ULOQ, LLOQ).<br>The concentration CV% based on the concentrations of 3 sets of samples should not exceed 20.0% (25.0% at ULOQ, LLOQ).<br>Total error (\|Bias%\|+CV%) ≤30.0% (ULOQ, LLOQ≤40.0%). |
| Inter-assay accuracy and precision | The concentration Bias% calculated from the mean concentrations of total runs should be within ±20.0% of the nominal value at each concentration level (±25.0% at ULOQ, LLOQ).<br>The concentration CV% calculated from mean concentrations of the total runs should not exceed 20.0% (25.0 % at ULOQ, LLOQ).<br>Total error (\|Bias%\|+CV%) ≤30.0% (ULOQ, LLOQ≤40.0%). |
| Assay Selectivity | For ≥ 80% of sources, unspiked matrix (0.00 µg/mL) should be BQL, and spiked samples should be ±25% at LLOQ with concentration CV% ≤25.0%, and ±20% at HQC with concentration CV% ≤20.0%. |
| Hook Effect and Dilution Linearity | For each diluted sample loading in the calibration curve range, the mean back-calculated concentration of 5 sets should be within ±20.0% of the nominal value and the related concentration CV% should not exceed 20.0%.<br>Hook effect: If the measured concentrations of the five high-concentration samples are within the range of quantification, the hook effect may be present. |
| Stability | For each concentration level, at least 67% of the three sets of the testing sample should be within ±20.0% of its nominal concentration with concentration CV% not exceed 20.0%. |
